# Dynamics of Macro- and Supramolecular Structures in the Bacterial Cytoplasm

**DOI:** 10.3390/microorganisms14102235

**Published:** 2026-10-03

**Authors:** Leon M. T. Dicks, Alfred Botha, Carolina Pohl

**Affiliations:** 1 Department of Microbiology, Stellenbosch University, Stellenbosch 7600, South Africa; abo@sun.ac.za; 2 Department of Microbiology and Biochemistry, University of the Free State, Bloemfontein 9300, South Africa; pohlch@ufs.ac.za

**Keywords:** bacteria, cytoplasm, particle dynamics, spatial compartmentalization

## Abstract

The bacterial cytoplasm is increasingly recognized as a dynamic, out-of-equilibrium environment in which biochemical reactions occur. Here, we discuss recent advances in our understanding of the generation of microscopic forces in the cytoplasm, particle dynamics, and spatial compartmentalization. Mounting evidence supports the opinion that the cytoplasm is fluidized by active forces generated by stochastic, energy-dependent interactions between metabolites and macromolecules. The forces promote protein mobility, conformational transitions, and biochemical interactions, thereby enhancing intracellular coordination. Bacterial cytoplasmic dynamics are best understood as an emergent property of collective, non-equilibrium processes, providing a unifying framework for intracellular organization and function and redefining the physical basis of bacterial cell biology. We explain how the excitation of nano- and macromolecules in the cytoplasm generates an out-of-equilibrium force that changes the cytoplasm from a glass-like to a semi-liquid phase. We critically evaluate proposed drivers of cytoplasmic fluidity, including nucleoid-associated processes and non-equilibrium active forces.

## 1. Introduction

The cytoplasm of bacteria lacks organelles, yet functions as a multi-part machine that responds to external stimuli. Densely packed macromolecular structures (e.g., granules, the nucleoid, proteins, enzymatic megacomplexes, and ribosomes), atoms, ions, and microcompartments create a highly constrained, microscopic meshwork that behaves like a glass-forming liquid in cells metabolically inactive or dormant [1,2,3,4]. In *Escherichia coli*, approximately 70% of the cytoplasmic space (1 μm^3^) is occupied by the 4.6 × 10^6^ bps nucleoid [5]. The average mesh size of an *E. coli* nucleoid is 50 nm [5], which implies that macromolecular assemblies exceeding 50 nm are excluded from the nucleoid. However, since the density of the nucleoid varies, smaller macromolecules may penetrate at random positions. The more compact the nucleoid, the smaller its mesh size (due to “shrinking”), and the more difficult it would be for proteins and other macromolecules to navigate a highly compressed DNA mesh [6]. The tight spacing slows down the diffusion of macromolecules, which may delay critical cellular processes such as transcription, replication, and DNA repair. Ribosomal subunits with Stokes radii of 15 to 20 nm and enzymes with a radius of 5 nm diffuse freely into the nucleoid [7,8,9,10]. This implies that 30 S and 50 S subunits diffuse freely, whilst fully assembled 70S ribosomes and polyribosomes are excluded from the nucleoid-occupied region [11]. The cytoplasm is a poor solvent, which supports genome compaction [5]. The *E. coli* nucleoid occupies the central region of the cell during slow growth but shifts toward the inner membrane (periphery) during rapid growth [5].

The behavior of macromolecular structures in the cytoplasm is explained by using the ‘spacers-and-stickers’ model, in which multivalent interactions between binding motifs (‘stickers’) are separated by flexible regions (‘spacers’) [12]. In the ‘associative polymer with percolation and phase separation’ model [13], the spatial orientation of the nucleoid is explained according to the formation of polymer networks (percolation) and the separation of phases. The movement of macromolecules such as ribonucleoproteins (BRs), RNA polymerases, and heat-unstable (HU) nucleoid-associated protein (NAP) (HUNP)–DNA condensates in the bacterial cytoplasm is best described by liquid–liquid phase separation (LLPS) [2,14,15,16].

In a dynamic (metabolically active) state, the cytoplasm becomes less glassy and more fluidic [2]. Large and small particles in the cytoplasm interact directly or group in vacuole-like structures called “cages” [1]. Larger particles that migrate in cages include protein condensates, aggregates (structured, dense solids such as fibrils and amorphous clumps), and agglomerates (loose, disordered clusters of particles); ribulose-1,5-bisphosphate carboxylase/oxygenase (RuBisCO); DNA and DNA-binding proteins from starved cells; polyphosphate biopolymers; ribonucleoprotein bodies; and glycogen [17]. Toward the end of the cell’s life, HUNP relaxes the highly compacted nucleoid, particles and cages rearrange, and open areas (“gaps”) form in the cytoplasm [1]. At this stage, the nucleoid divides, and cell division starts [2,5].

Protein-bound cages contribute to cytoplasmic viscosity [18]. In an eukaryote study using HeLa, VMKO HeLa, and MDCK cells, Ebata et al. [19] concluded that the cytoplasm of metabolically active cells exhibited a single-exponent power-law rheology, expressed as *G* = G_1_(−*iω*)^0.5^ (*G* = complex shear modulus, combining elastic storage and viscous loss moduli; G_1_ = material-specific scale factor or amplitude coefficient; −*i* = imaginary unit rotation representing phase lag; *ω* = angular frequency of the applied oscillation; 0.5 = the power-law exponent, representing critical jamming behavior). This is also observed in sheared, concentrated, starlike micelles [20] and dense, motile bacterial suspensions in a nutrient-exchange chamber [21]. Further research is required to confirm this behavior in intact viable bacterial cells.

Several studies have suggested that ribosomes [22,23], pH [24], and ATP [1] control cytoplasmic rheology. However, more recently, Losa et al. [25] demonstrated that cytoplasmic rheology in *E. coli* is controlled by protein agglomerates. The authors tracked a 40 nm-sized encapsulin-based fluorescent particle with photoactivated light microscopy and observed that some amino acid metabolism proteins form clusters. Electron microscopy showed that these proteins can form amorphous agglomerates at physiological concentrations in vitro due to their high hydropathy, which also confers low disorder and compactness. When highly expressed, protein agglomerates obstruct the diffusion of nanoparticles. Membrane-bound organelles are rare and are only found in planctomyces (anammoxosomes), cyanobacteria (thylakoids), and magnetotactic bacteria (magnetosomes) [26,27,28]. The rheology of the cytoplasm may also be altered by proteinaceous membrane-less microcompartments, also known as metabolosomes. Examples include carboxysomes encapsulating CO_2_, and Pdu, Etu, and Eut microcompartments containing enzymes that metabolize 1,2-propanediol, ethanol, and ethanolamine, respectively [29]. Crystals of magnetite (Fe_3_O_4_) and greigite (Fe_3_S_4_) found in magnetotactic bacteria (*Magnetospirillum*, *Magnetovibrio* and *Magnetococcus*) are enclosed in nano-magnetosomes [28].

This review discusses how particle dynamics and spatial compartmentalization influence the formation of microscopic forces in the cytoplasm. We identify the drivers of cytoplasmic fluidity responsible for the generation of an out-of-equilibrium force that changes the cytoplasm from a glass-like to a semi-liquid phase. The review does not cover interactions between proteins and the cell membrane. The behavior of proteins near cell structures is influenced by electrostatic and/or hydrophobic interactions, as well as repulsive volume-exclusion interactions, and warrants a separate discussion. We acknowledge that interfacial forces such as hydrophobic, electrostatic, and hydrogen interactions may act as physical catalysts by lowering the energy barrier required for molecules to self-assemble. This may cause conformational shifts in proteins and may expose intrinsically disordered regions (IDRs) that act as drivers of condensate formation. These interfacial forces are mostly associated with membranes and nucleic acid scaffolds, which are not discussed in this review.

## 2. Physicochemical Properties of the Cytoplasm

**Figure 1:**
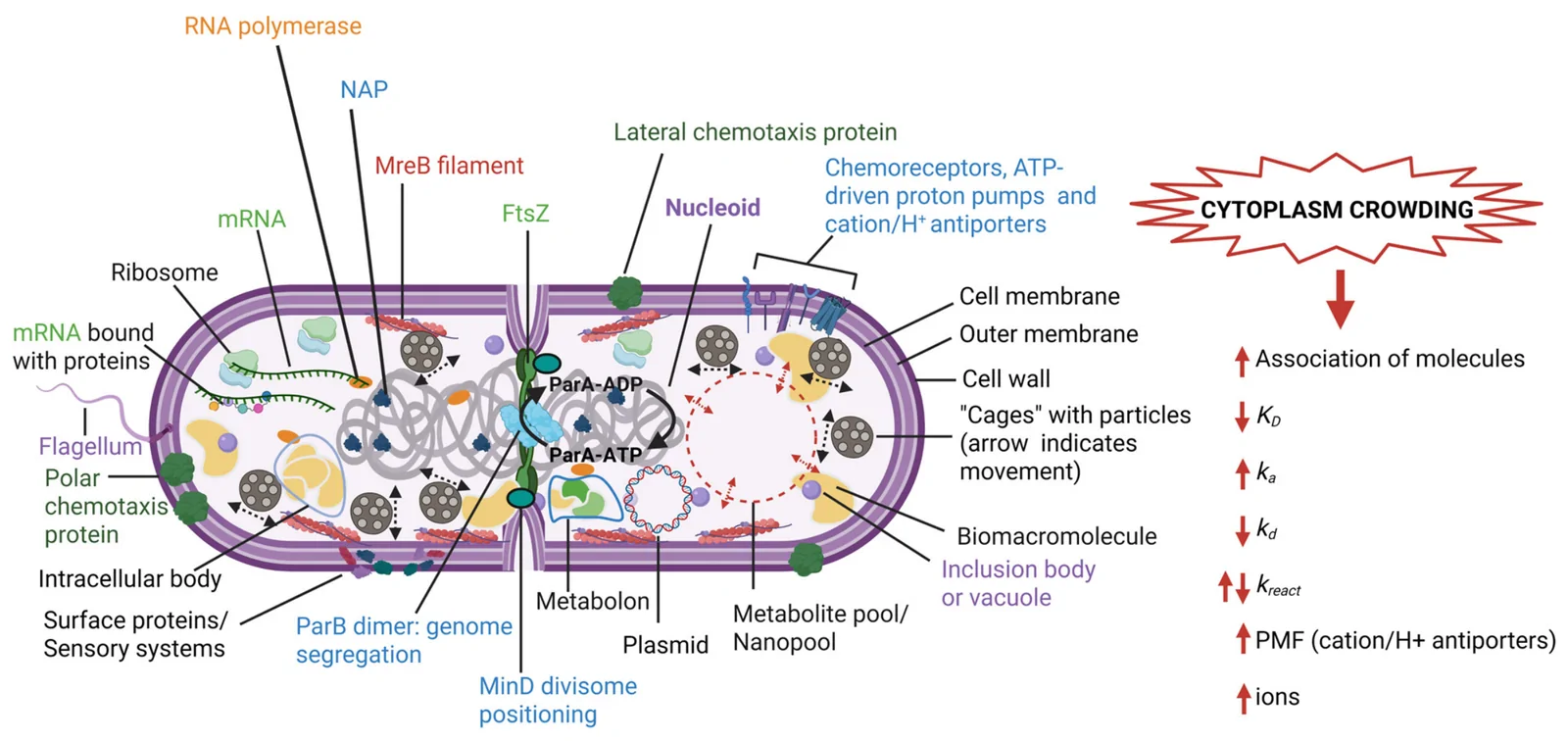
Gram-negative bacterial cell with spatially organized intracellular (cytoplasmic) structures. The nucleoid, plasmids, inorganic and organic molecules, proteins, ribosomes, enzymatic megacomplexes, granules, and microcompartments give the cytoplasm a glass-like appearance. Metabolic processes fluidize the cytoplasm and separate particles, changing the glass-like appearance from semi-solid to semi-liquid. Particles in microcompartments (grey spheres) rearrange (indicated by black dashed arrows) to form open areas (“gaps”) in the cytoplasm. The movement of particles generates dynamic heterogeneity within the cytoplasm. The protein ParB binds to the *parS* region of the bacterial genome. When the ParB-genome complex encounters ParA-ATP, ParB acts as a GAP (GTPase/ATPase activating protein) and forces ParA to hydrolyze ATP into ADP. The ParA/MinD ATPase proteins regulate chromosome segregation and cell division. The exchange between ADP and ATP in ParA “shifts” the genome towards opposite poles of the cell, forming a “wave”-like movement. MreB, a helical actin homolog in the cytoplasm, positioned underneath the cell membrane, maintains rod shapes by connecting inner-cytoplasmic filaments to cell-wall synthesis machinery across the membrane. RNA polymerases and other molecules form dense regions, a process known as liquid–liquid phase separation (LLPS). mRNAs are shown as free or as bound to ribosomes or proteins. Biomacromolecules (indicated in yellow) contribute to cytoplasmic crowding and can aggregate to form intracellular bodies. Open spaces (“gaps”) in the cytoplasm serve as metabolite pools (red dashed circle) and allow molecules to move in and out of the pool (red arrows). The image on the right summarizes the changes in forces during crowding. Red arrows pointing up = increase in activity, arrows pointing down = decrease in activity. *K_D_* = dissociation constant, *k_a_* = association rate constant (a higher *k_a_* turns the reaction “on”), kd = dissociation rate (a low kd turns the reaction “off”), *k_react_* = reaction rate constant, PMF = proton motive force, NAP = nucleoid-associated protein. Modified from a figure published by Dicks et al. [2]. Created in Biorender. L.M.T. Dicks. https://app.biorender.com/illustrations/6aa1531d807da3ef72eb5a7a, assessed on 28 September 2026.

Densely packed macromolecules (referred to as crowding) lead to the formation of large open areas/gaps (Figure 1) [1,2,3,4] and give the cytoplasm a glass-like appearance [1,2]. Crowding increases the chemical activity (effective concentration) of molecules (estimated at 25 to 40% of the cytoplasmic volume), thereby thermodynamically favoring and enhancing molecular association and biochemical reactions [30] (Figure 1). At the same time, an increase in the affinity between an enzyme and its substrate (ES complex) decreases the dissociation constant (*K_D_*) (Figure 1) [31,32]. A lower *K_D_* indicates higher binding affinity (a stronger interaction between a protein and its target). *K_D_* is inversely proportional to the association rate constant (*k_a_*, the “on” rate in Equation (1)), assuming that the dissociation rate constant *k_d_* (the “off” rate) is held constant. At higher *k_a_* (faster turning “on” of reactions, i.e., binding of substrates) or a lower *k_d_* (slower turning “off” of reactions, i.e., slower rate at which the ES complex dissociates and does not react), molecular dissociation decreases and binding affinity increases. At lower *K_D_*, the ES complex is highly stable. This stability lowers the baseline free energy (*G*) of the complex deep into a thermodynamic valley, also described as a deep thermodynamic pit or “substrate sticky trap”. Tight binding to the initial substrate does not inherently (automatically) increase the chemical conversion rate constant (*k_react_* in Equation (2)). Extremely stable enzyme-substrate complexes raise the overall activation energy barrier (Δ*G*^‡^) because the energetic climb out of the “sticky trap” to the transition state becomes much steeper, potentially slowing down the catalytic step. For reactions driven by a high *k_a_*, e.g., covalent inhibitors or proximity-induced reactions, a low *K_D_* does not alter the intrinsic chemical reactivity (*k_react_*) but drastically increases the overall rate of product formation by driving the equilibrium forward, ensuring that the enzyme is almost always saturated and driven toward the reactive complex.

(1) $$K_{D}\;=\;\frac{\mathrm{kd}}{\mathrm{ka}}$$

(2) $$k_{a}\alpha \frac{K_{D}}{k_{\mathrm{react}}}$$

The overall or apparent association rate constant (*k_a_*)is related to the diffusion-limited rate constant (*k*_D_) and the intrinsic chemical reaction rate constant (*k_react_*) by the steady state relation (Equation (3)). With an increase in *k*_a_, the sum of the inverse values of the rate constant under diffusion control (*k*_D_) and *k*_react_ decreases. This means the combined resistance (1/*k*_D_ + 1/*k*_react_) decreases. If *k*_react_ is slow or small (*k_react_* << *k*_D_), the chemical step becomes the bottleneck of the system, and the overall rate simplifies to *k_a_* ≈ *k_react_*. Holding reactants in an optimal spatial orientation drastically reduces the activation entropy barrier and increases the intrinsic value of *k_react_*, thereby accelerating the overall process.

(3) $$k_{a}\;=\;\frac{k_{D}k_{\mathrm{react}}}{k_{D}+k_{\mathrm{react}}}$$

In a reduced volume (crowded space) with high viscosity, fewer separate undissociated reactions form, and the activation energy barrier is much lower. Thus, molecular motion is slowed in a higher-viscosity medium, and fast reactions that depend on random interactions are hindered [33,34,35]. The incredibly tight binding between ribonuclease (RNase) and the RNase inhibitor (RI) protein is an example of an enzyme-inhibitor complex with a low KD (approximately 10^−15^ M). At this low *K_D_*, the RI protein wraps around RNase and serves as a biological “kill-switch”, protecting RNA from destruction.

**Figure 2:**
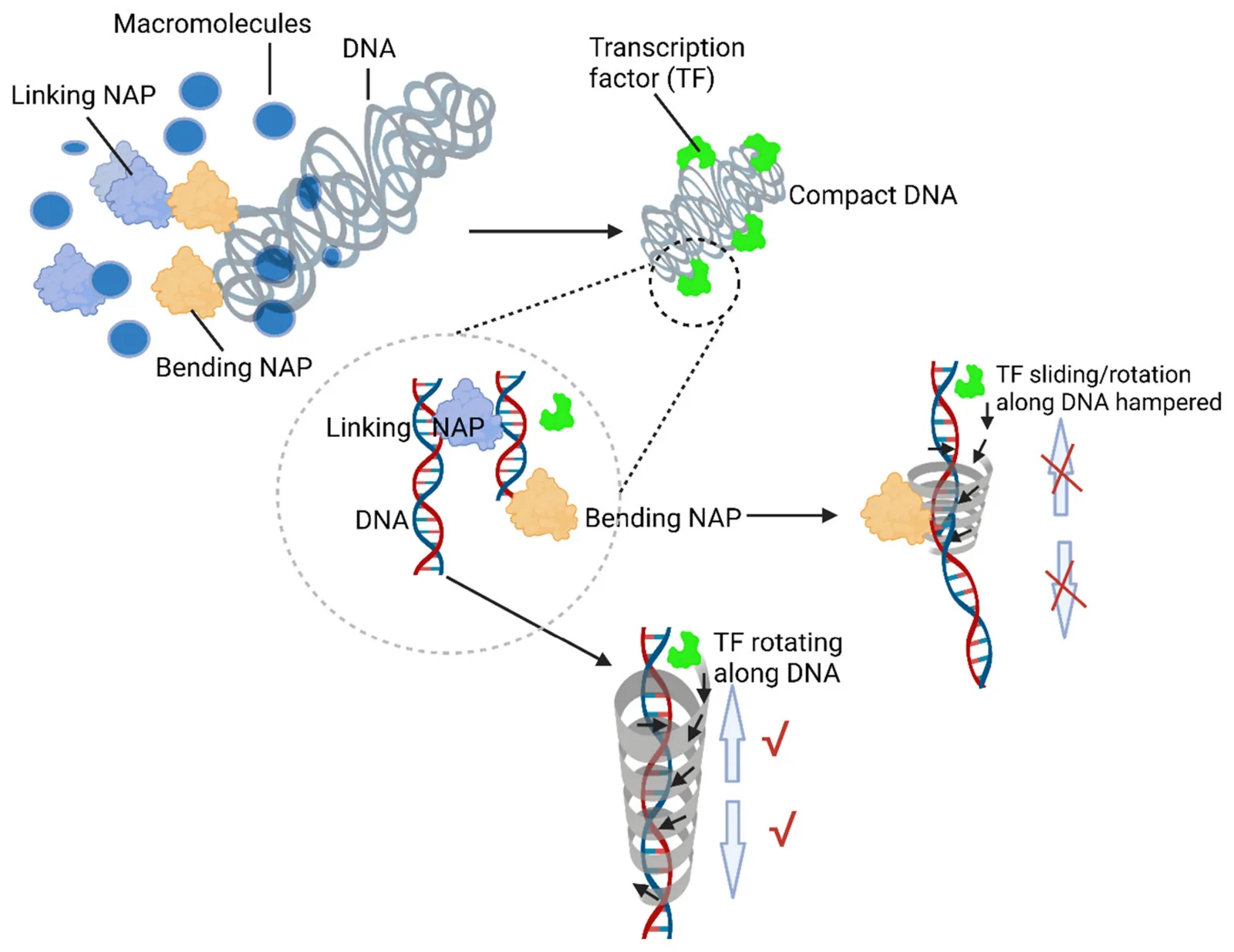
The influence of macromolecular crowding on gene regulation kinetics. Linking nucleoid-associated proteins (NAPs) link DNA strands (**left**), whilst bending NAPs bend the DNA and force the nucleoid into a compact conformation. This increases transcription factor (TF) binding to the nucleoid. The TFs rotate along the grooves of the DNA molecule, described as facilitated diffusion/sliding. Sliding is hampered by bending NAPs (**right**) but facilitated in the absence of NAP binding (**bottom**). The overall target search (TF binding to DNA) is accelerated by crowding (a smaller reaction volume). Created in Biorender. L.M.T. Dicks. (2026). https://app.biorender.com/illustrations/6aa3c30943e22a208dc4903b, assessed on 28 September 2026.

Macromolecular crowding affects gene expression by altering the diffusion constants of transcription factors (TFs), as illustrated in Figure 2 [36]. Nucleoid-associated proteins (NAPs) bind to the genome and alter its three-dimensional structure, forming a compact nucleoid. Transcription factors (TFs) bind either specifically or non-specifically (randomly) to the nucleoid. The strong association with DNA causes the TFs to rotate more slowly along the grooves of the DNA molecule, a process referred to as facilitated diffusion/sliding (Figure 2). The physical distortion of DNA by NAPs disrupts the continuous helical path required for the TF to glide smoothly from one base pair to the next, severely shortening its one-dimensional sliding length (Figure 2) and causing premature dissociation. The rotational motion is associated with friction, proportional to the volume of the protein, and slows down the movement of TFs [37]. One-dimensional diffusion coefficients for TFs are substantially (two orders of magnitude) smaller than three-dimensional diffusion constants, suggesting that sliding down the nucleoid occurs much more slowly than diffusion through the cytoplasm. Norred et al. [38] have shown that macromolecular crowding influences the rate of gene expression and have described how mRNA and proteins are produced in bursts, a phenomenon associated with cytoplasmic noise (increased protein levels). This occurs at modest changes in mRNA levels, attributed to the non-homogeneous spatial distribution of mRNA in a crowded cytoplasm [38]. Thus, mRNA spatial noise leads to large temporal fluctuations in protein levels. Our understanding of the interplay between macromolecular crowding, gene expression, and spatial distribution (diffusion) of mRNA is limited and requires in-depth research. A better understanding of these processes will facilitate research in cell-based and synthetic biology.

The cytoplasm of metabolically active cells is in a homeostatic state, i.e., the physical and chemical conditions, such as macromolecular crowding, pH, ionic strength, and turgor pressure, are carefully controlled to maintain balance (reviewed by Poolman [39]). Protons, which participate in biochemical reactions as reactants and/or regulators of enzyme activity, serve as a source of electrochemical energy (proton motive force, PMF) and may influence LLPS (Figure 1). PMF is influenced by the membrane potential (ΔΨ), which is typically negative in the cytoplasm, and the difference in cellular and external pH (ΔpH) (Equation (4)).

PMF = ΔΨ-ZΔpH(4)

**Figure 3:**
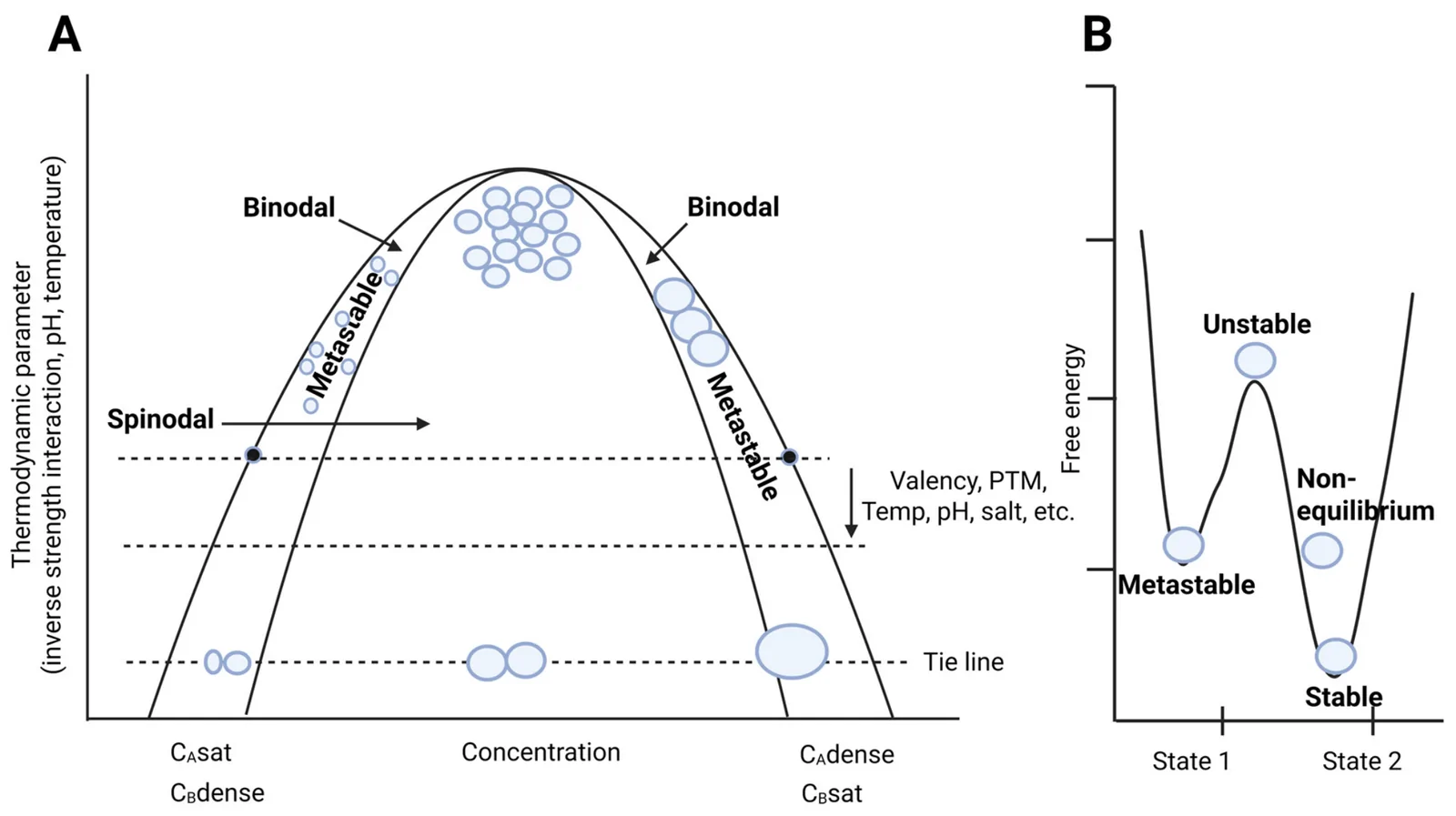
The principles of liquid–liquid phase separation (LLPS) in a binary system. (**A**): Graphical representation of the phases. (**B**): Changes in energy drive the stability/instability of thermodynamic systems. In graph (**A**), C_A_ = concentration of molecule A and C_B_ = concentration of solvent/molecule B (in this case, the cytosol). At very low concentration, molecule A dissolves in B, and the system remains as a stable, single-phase homogeneous solution. As the concentration of molecule A increases, it reaches a point where the solubility of A in B saturates (C_A_sat). When this point is crossed, condensates form as follows: Molecules A and B demix into two liquid phases at the binodal (coexistence) line. However, for this to happen, a trigger or nucleation event must occur in the region between the spinodal and binodal lines, and a metastable structure forms. As the concentration of molecule A increases, and the spinodal line is reached, the trigger is no longer necessary, as the mixture is unstable and spontaneously separates into two coexisting liquids or phases, referred to as the spinodal decomposition. With a further increase in A (denoted as C_A_dense), the upper limit of the binodal line is reached, and the mixture becomes energetically favorable, this time predominantly A. The tie line in the graph connects points of equal chemical potentials. Along this line, the size of the droplets increases, but the concentration within the droplet remains the same, because the partial molar free energy is equal. To change the molar fractions of C_A_sat and C_A_dense, the thermodynamic parameter must be adjusted. The lower the Csat (concentration of saturation), the stronger the driving forces of phase separation. At the critical point, the mixture is more favorable than the two existing phases. Temp = temperature, PTM = post-translational modifications. Created in Biorender. L.M.T. Dicks. (2026). https://app.biorender.com/illustrations/6ab3a10af477a4dcfbe20af5, assessed on 28 September 2026.

The symbol “Z” denotes the relation between the gas constant (R), absolute temperature (T), and the Faraday constant (F), formulated as 2.3 RT/F [39]. Thus, to maintain a constant PMF, cells need to maintain electron charge (ion levels), temperature, and regulate thermodynamic and kinetic forces. PMF is generated by electron transport, light-driven proton translocation, ATP-driven proton pumps, or coupling of electrogenic transport to a metabolic reaction [40]. Each of these mechanisms increases the internal pH, which is kept in balance by cation/H^+^ antiporters, anion/H^+^ antiporters, and metabolite decarboxylation [39]. Protons are pumped out by respiration or other mechanisms and pumped back into the cell by PMF-consuming processes such as ATP synthesis or nutrient uptake [39]. Ionic strength influences the binding of proteins to nucleic acids, protein aggregation, the activity of enzymes, the structure of intrinsically disordered proteins (IDPs), ion channels, transporters, and phase separations [41,42,43]. Changes in the physical interactions between macromolecules in a crowded cytoplasm, e.g., due to increased ionic strength or lower pH, may affect the translational diffusion of proteins [44] and compromise proteome stability [45]. Proteins or protein domains that unfold as a result of, e.g., heat stress may represent IDPs and increase the viscosity of that specific area in the cytoplasm, causing the proteins to become entangled [46]. Changes in temperature, ionic strength, and post-translational modifications (PTMs) alter the critical threshold for phase separation (Figure 3). The threshold concentration of biomolecules required for phase separation is indicated by the dotted line in Figure 3. In the binodal curve, the boundary line represents the physiological conditions (e.g., temperature, pH, ion concentrations) at which the cytoplasm is saturated with biomolecules (C_sat_). Below C_sat_, the system is in a homogeneous, single-phase region. The phase coexistence region is the area above (or inside) the binodal curve where the solution splits and two distinct phases exist simultaneously (Figure 3). Even dispersion allows proteins to associate at much lower concentrations. Above C_sat_, the cytoplasm becomes supersaturated and spontaneously splits into two phases (depicted in Figure 3 as the dilute and dense phases, respectively). In standard LLPS, both phases are entirely liquid. The separation of biomolecules into different phases alters the crowdedness of the cytoplasm. The dense phase behaves like a highly viscous material that may restrict the diffusion of large particles while accelerating localized reactions. Proteins in the dilute phase will attract each other, nucleic acids, and other macromolecules in a nonspecific manner. Multivalent or non-specific interactions between molecules in a single homogeneous solution cause the separation into two phases [47]. In the dense phase, protein associations are enhanced via volume exclusion. The diluted phase is depleted of protein, relative to the total composition. Macromolecules may also be dispersed via substrate channeling, mediated by metabolons (transient, noncovalently assembled enzyme clusters) that enable direct metabolite transfer between active sites. Examples are intramolecular tunnels (e.g., tryptophan synthase), electrostatic channels (e.g., between malate dehydrogenase and citrate synthase), chemical swing arms (e.g., pyruvate dehydrogenase), cluster channeling (e.g., purinosomes), and glycolytic bodies (reviewed by Fukuhara et al. [48]).

The behavior of proteins in a crowded cytoplasm was originally thought to be dictated by diffusion according to the Stokes–Einstein (SE) relation (τ∝^η^/_T_), where τ is the diffusion time, η is the viscosity of the medium, and T is the experimental temperature [49,50]. The classical SE equation assumes that a molecule behaves as a rigid sphere diffusing thermally and passively through a homogeneous, continuous fluid of a fixed viscosity (η). Recent findings have shown that the cytoplasm behaves as a complex, non-equilibrium material rather than a simple aqueous solution. Macromolecular crowding forces the cytoplasm to behave similarly to a highly concentrated colloidal fluid or a glass-forming liquid near its vitrification (glass transition) point. At these densities, molecules undergo “glassy dynamics,” in which movement is severely restricted by local “cages” formed by neighboring molecules. Transport is characterized by sudden hopping motions rather than continuous Brownian diffusion, thereby violating the SE relationship. The translational diffusion coefficient (D) of proteins (study performed on *Escherichia coli*) is determined by the mass of a protein (polypeptide), multiplied by the oligomeric state, and not abundance [8,51]. Furthermore, D = αMβ, where M is the complex mass and α and β are fitting parameters. In the SE equation, β would be −0.33 for globular proteins that do not interact with each other. Instead, β was determined to be −0.6 for the diffusion of proteins in the cytoplasm of *E. coli* [8,52]. This implies that the dependence on molecular mass is much stronger than originally anticipated in the SE equation (due to macromolecular crowding). The observed molecular-mass dependence deviates significantly from standard SE scaling, indicating that the bacterial cytoplasm exhibits anomalous, crowded-diffusion characteristics. While this fractional scaling highlights the complex, non-Newtonian environment of the cell interior, further micro- or macro-rheological measurements mapping stress against shear rate are required to definitively characterize the bulk fluid type. A modified version of the SE equation was proposed (Equation (5)):

(5) $$D\;=\;\frac{k_{B}T}{{6\pi \eta }_{\mathrm{MW}}r}$$

where *η_MW_* represents the perceived viscosity (apparent thickness or resistance to cytoplasmic mobility) as a function of the molecular weight. The perceived macromolecular viscosity changes visually and varies from 9.9 cP to 18.1 cP (centipoise) for proteins of 26 kDa to 318.9 kDa [53]. Deviation from the SE equation was confirmed by using fluorescence recovery after photobleaching (FRAP) and nuclear magnetic resonance (NMR) [54]. If diffusion is related to viscosity, as shown in the fractional Debye-Stokes-Einstein (FDE) equation τ∝ (^η^/_T_)^α^ (τ = time for a molecule to structurally rearrange or rotate, η = the fluid’s internal resistance to cytoplasmic diffusion, T = absolute temperature, and α = the fractional exponent/scaling parameter), and α is less than 1.0, diffusion is inconsistent. An α value less than 1.0 is typical for time-fractional FDEs. It models a system with “memory effects”, i.e., where the future state depends on its historical trajectory, or when particles diffuse more slowly than normal [55]. Proteins in complex fluids near the glass transition have α-values of 0.7 to 1.0, depending on the degree of decoupling [56]. By using fluorescence correlation spectroscopy (FCS) and a FRET-based biosensor that detects the transfer of Förster resonance non-radiative energy from an excited donor fluorophore to a nearby acceptor fluorophore, Junker et al. [57] have shown a consistent negative deviation from the SE relation as the molecular weight of macromolecules increases. The diffusion of proteins in the mass range of 26–319 kDa correlates with the average 50 nm mesh size of the *E. coli* genome [5]. This means proteins with a Stokes radius up to 5 nm may not be affected by the genome’s position in the cytoplasm. Proteins at the poles of *E. coli* diffused more slowly than those in the rest of the cytoplasm, as shown by fusing native *E. coli* proteins to a photoswitchable fluorescent protein, mEos3.2 [58]. Computer simulations demonstrated that the decrease in protein diffusion exceeds the constraining effects of the cell membrane. The accumulation of proteins most likely begins with genetic mutations, environmental stress (e.g., heat or oxidation), or aging. Misfolded proteins are cleared by chaperones that refold proteins and the proteasome. In bacteria, damaged proteins accumulate at cell poles [58]. As expected, the rate at which cytoplasmic proteins diffuse varies among species. An increase in macromolecular crowders may alter protein conformations from a relaxed, unfolded state to compact, quasi-spherical forms [59]. Proteins with no stable three-dimensional form under physiological conditions (referred to as intrinsically disordered proteins, IDPs) may become compacted in a crowded cytoplasm. Examples are carboxyamidated RNase T1 in the presence of dextran [60] and flavodoxin, and apoflavodoxin, and *Borrelia burgdorferi* VisE in the presence of ficoll-70 k (ficoll PM 70), a neutral, highly branched, hydrophilic synthetic polymer of approximately 70 kDa [61]. Although ficoll PM 70 is not naturally present in cells, the in vitro experiments performed by Perham et al. [61] indicated that the native states of proteins (at least for small proteins with marginal stability) may differ in the presence of macromolecular crowding agents and imply that the functional structures of proteins with low intrinsic stability may differ in vivo from proteins in dilute buffers.

Macromolecular crowding helps maintain the three-dimensional structure of DNA and proteins, regulates the kinetic and thermodynamic behavior of enzymes [62,63], facilitates the binding of molecules to ligands, the formation of insoluble aggregates, and the compaction/folding of proteins [4,64,65]. Most of these findings were based on in vitro studies and need to be confirmed in vivo. Chromosome segregation and cell division are regulated by the ParA/MinD ATPase proteins, as shown in Figure 1 (reviewed by Dicks [2]). ParA orchestrates the segregation of genomic DNA, and MinD positions the divisome (the protein complex responsible for bacterial cell division) at the center of the cell [66]. Protein ParB binds to *parS*, a centromere-like site on the genome, and forms a large partition complex. ParB stimulates the ATPase of ParA and the ParA-ADP complexes. The ParA-ADP complex dissociates from the genome and is replaced by the next available ParA-ATP complex. This continuous cycle of ParA-ADP and ParA-ATP “shifts” the genome toward areas of higher ParA-ATP density located at opposite poles of the cell, as shown using a mathematical motion model combined with epifluorescent microscopy of *E. coli* cells [67]. Genome shifting forms a “wave”, as concluded from high-throughput imaging analysis and molecular computational modeling performed on *E. coli* [68]. MreB maintains rod shapes by connecting inner-cytoplasmic filaments to cell-wall synthesis machinery across the membrane [69]. The tubulin protein FtsZ, positioned at the center of the cell, forms the Z-ring and binds to the inner surface of the cytoplasmic membrane [70]. Other cell particles are displaced in an equally well-controlled manner.

## 3. Drivers of Cytoplasm Dynamics

Metabolic activities fluidize the cytoplasm and allow microcompartments to group in overcrowded regions (nanopools), indicated in Figure 1. Regions in the cell with nanopools are metabolically more active [71], which implies that the rearrangement (turnover) of nanopools would promote the displacement of microcompartments over longer distances in the cytoplasm. Meng et al. [71] simulated a model system of bacterial cytoplasm based on experimental data of *E. coli* and argued that the positioning of microcompartments, ranging from subnanometers to micrometers in size, and differences in their diffusion rates in the cytoplasm may hold the key to movement (cytoplasmic fluidity). In a dynamic state (metabolically active cytoplasm), particles are energized by the random “moving” of ATP, amino acids, and other metabolites [72,73,74,75]. Parry et al. [1], however, argued that DNA-related processes (e.g., transcription), the displacement of the nucleoid, and the turnover of cytoplasmic content due to metabolic processes (by definition, also microcompartments) are not the driving force behind cytoplasmic fluidity. This conclusion was based on tracking proteins, plasmids, storage granules, and particles of different sizes using green fluorescent protein (GFP) labeling. The findings of Parry et al. [1] aligned with the view formulated by Monti et al. [76], although this group’s research was based on a whole-brain model and is not directly applicable to bacterial cells. Despite these cross-system analogies, findings from both scientific groups, Parry et al. [1] and Monti et al. [76], have shown that collisions between particles, or microcompartments, occur randomly and thus out of equilibrium. For the fluctuation–dissipation (FD) theorem to apply, particles in the cytoplasm must be in equilibrium, as concluded using a model-based nonequilibrium FD theorem [76]. In the absence of FD, the internal fluctuations (e.g., Brownian motion), are not identical to the dissipation of energy in a weak force or agitation (with reference to conditions in the cytoplasm). Put differently, the fluctuations of particles must correspond to fluctuations in their energy to generate a fluidic cytoplasmic force that would force interactions between particles. These findings describing particle behavior need to be confirmed by in vivo experiments on viable bacteria. For further reading on the FD theorem, the reader is referred to Monti et al. [76].

In modeling a bacterial cell, Meng et al. [71] have shown that cytoplasmic dynamics arise from a combination of metabolic processes. The authors determined the magnitude of the active force (F) in the cytoplasm to be 0.57 pN (pico Newton). This, they argue, is generated by the combined collisions between small molecules, amino acids, proteins, and ions that are out of equilibrium. Based on the size of a typical protein (10 nm, as defined by the authors), the passive thermal force (*F* = *k_B_T*/*a*) arriving from the ambient thermal energy (*k_B_T*) of a protein with a size (*a* value) of 10 nm relates to 0.4 pN. *k_B_* is the Boltzmann constant [≈ 1.38 × 10^−23^ J/K (Joules per Kelvin)], and *T* is the temperature in K. While the baseline calculation uses a thermal framework, the actual bacterial cytoplasm is a non-equilibrium system. Continuous injection of metabolic energy (e.g., ATP hydrolysis) creates “active noise” or nonthermal fluctuations. This out-of-equilibrium activity effectively boosts the fluctuating forces from the baseline thermal level (0.4 pN) up to the actively enhanced level (0.57 pN). This extra physical agitation may help the cell accelerate intracellular particle transport, fluidize cytoplasm, and prevent cellular components from becoming trapped in local energy minima. The Boltzmann constant relates the average microscopic thermal energy of particles to the macroscopic thermodynamic temperature of a system [77]. According to Meng et al. [71], a minimal mechanical force of 0.4 pN is sufficient to counteract ambient thermal fluctuations in the cytoplasm and to allow signaling proteins to move in a specific direction or change the shape of large molecular structures, thereby triggering cellular signals.

## 4. Size Matters in Diffusion

The rate at which macromolecules diffuse across the cytoplasm determines the speed or frequency of collisions. This is true when reactions are diffusion-limited, i.e., when the association rate constant (*k*_a_) depends only on the translational diffusion coefficient (D). However, the *k*_a_ of a protein diffusing in the cytoplasm with D = 10 μm^2^/s and reacting with another molecule is approximately 10^8^ M^−1^/s (M = molar). Since most proteins have catalytic regions and are not reactive across their entire surface, a more realistic diffusion-limited *k*_a_ would be 10^5^ to 10^6^ M^−1^/s [75]. The Barnase–Barstar protein pair of *Bacillus amyloliquefaciens* has a *k*_a_ of 10^8^ to 10^10^ M^−1^/s, which is beyond the diffusion limit. Based on theoretical models [78,79] and fluorescence spectra [80], the interaction between these proteins is mediated by electrostatic attraction, enabling ribonuclease binding to the inhibitor protein at orders of magnitude above the non-electrostatic diffusion limit. Other diffusion-limited protein reactions with high *k*_a_ include the ternary complex of aminoacyl-tRNA, EF-TU, and GTP that interacts with the ribosome, known as tRNA selection or decoding [81,82]; proteins in low copy numbers and with longer distances to cover; and proteins binding to membranes or other large structures (e.g., the Min oscillation system) [83].

Protein–protein interactions, such as those between cationic Barnase and anionic Barstar, are rare in bacteria. Only 35 proteins in *E. coli* have a net surface charge >+10. Of these, 18 are ribosomal, 14 are DNA/RNA-associated, and 3 have unknown functions [53]. It thus seems that the bacterial proteome is largely anionic, thereby supporting rapid diffusion through the cytoplasm. It also implies that endosymbionts with highly cationic proteomes have evolved specialized mechanisms to avoid slow diffusion and impair ribosomal function. As crowding increases, the *D* of macromolecules decreases by orders of magnitude, and many reaction-limited interactions between macromolecules become diffusion-limited. Small molecules, such as sugars trapped in supramolecular protein aggregates, are not severely affected by osmotic upshifts and diffuse throughout the cytoplasm, even at NaCl concentrations of 1 M or higher [50,84]. The cytoplasm thus acts as a molecular sieve under high and low osmotic stress, but with different mesh sizes. This allows the rapid diffusion of ions, small metabolites, and signaling molecules across a crowded cytoplasm.

## 5. Conclusions

We argue that cytoplasmic fluctuations are unlikely to be caused by nucleoid displacement or metabolic processes, and are more likely due to particles within microcompartments that move randomly in a non-equilibrium state and are localized in nanopools at the two poles of the cell. Based on models and in vitro studies, protein movement causes cytoplasmic fluidization and increases biochemical reactions, which may increase metabolic activity. In addition, particles may be energized by the random “moving” of ATP, amino acids, and other metabolites. Concluded from these findings, macromolecular crowding is a key contributor to intracellular complexity and plays a major role in regulating protein–protein, protein–nucleic acid, and protein–lipid interactions. The spatial orientation of biomolecular condensates is regulated by supramolecular structures such as DNA and RNA, as well as by PTMs and the cell membrane. The exact mechanism by which PTMs regulate phase separation must be confirmed. The size of the nucleoid-free space in the cytoplasm and the concentration of proteins are likely determining the size of a condensate. Further research is required into the ultrastructure of biomolecular condensates and the organization of their components, especially in heterotypic condensates. We do not know to what extent the stoichiometry of complexes in a semi-solid (dense, highly viscous, glass-like) cytoplasm changes during turnover to a semi-liquid phase. Examining crowding effects on the structure, function, and regulation of biomolecular condensates is challenging. Not all biomolecular condensates are manipulated by macromolecular crowding. In some cases, condensation is triggered by ionic strength, pH, ATP, and membrane surfaces. Many questions remain unanswered or not fully understood, e.g., the effect of ribosomes on protein mobility, the effect of protein levels on reaction rates and diffusion coefficients, the influence of metabolic conditions on LLPS formation, differences in the mobility of old and new proteins at cell poles, conditions or reactions affecting the compaction of the nucleoid, factors determining the size of biomolecular condensates, and the role of nucleotides in the assembly of condensates.

## Data Availability

No new data were created or analyzed in this study. Data sharing is not applicable to this article.
